# A novel and fully automated mammographic texture analysis for risk prediction: results from two case-control studies

**DOI:** 10.1186/s13058-017-0906-6

**Published:** 2017-10-18

**Authors:** Chao Wang, Adam R. Brentnall, Jack Cuzick, Elaine F. Harkness, D. Gareth Evans, Susan Astley

**Affiliations:** 1 Centre for Cancer Prevention, Wolfson Institute of Preventive Medicine, Queen Mary University of London, Charterhouse Square, London, EC1M 6BQ UK; 2 Centre for Imaging Science, School of Health Sciences, University of Manchester, Stopford Building, Oxford Road, Manchester, M13 9PT UK; 3 Department of Genomic Medicine, University of Manchester, St Mary’s Hospital, Manchester, M13 9WL UK

**Keywords:** Breast density, Texture, Digital mammogram, Risk prediction, Breast cancer

## Abstract

**Background:**

The percentage of mammographic dense tissue (PD) is an important risk factor for breast cancer, and there is some evidence that texture features may further improve predictive ability. However, relatively little work has assessed or validated textural feature algorithms using raw full field digital mammograms (FFDM).

**Method:**

A case-control study nested within a screening cohort (age 46–73 years) from Manchester UK was used to develop a texture feature risk score (264 cases diagnosed at the same time as mammogram of the contralateral breast, 787 controls) using the least absolute shrinkage and selection operator (LASSO) method for 112 features, and validated in a second case-control study from the same cohort but with cases diagnosed after the index mammogram (317 cases, 931 controls). Predictive ability was assessed using deviance and matched concordance index (mC). The ability to improve risk estimation beyond percent volumetric density (Volpara) was evaluated using conditional logistic regression.

**Results:**

The strongest features identified in the training set were “sum average” based on the grey-level co-occurrence matrix at low image resolutions (original resolution 10.628 pixels per mm; downsized by factors of 16, 32 and 64), which had a better deviance and mC than volumetric PD. In the validation study, the risk score combining the three sum average features achieved a better deviance than volumetric PD (Δχ^2^ = 10.55 or 6.95 if logarithm PD) and a similar mC to volumetric PD (0.58 and 0.57, respectively). The risk score added independent information to volumetric PD (Δχ^2^ = 14.38, *p* = 0.0008).

**Conclusion:**

Textural features based on digital mammograms improve risk assessment beyond volumetric percentage density. The features and risk score developed need further investigation in other settings.

**Electronic supplementary material:**

The online version of this article (doi:10.1186/s13058-017-0906-6) contains supplementary material, which is available to authorized users.

## Background

Mammographic density is a term used to describe whiter regions of images that reflect the amount of fibroglandular as opposed to fatty tissue in the breast. Mammographic density is a well-established risk factor for breast cancer [1]. One well-studied measure of breast density is the percentage of the breast area that is opaque, often referred to as percent density (PD). In addition to area-based PD, volumetric measures have been developed to make use of the greyscale pixel values, without thresholding. It has been estimated that 16% of all breast cancers and 26% of breast cancers in women aged 55 years or less are attributable to breast density over 50% [2]; women with PD over 75% have been consistently reported to be at a fourfold to sixfold higher risk of developing the disease than women of similar age with little or no dense tissue [3]; and PD has been described as a risk factor that is the most significant after age [4].

While PD is an important risk factor, it is likely that characteristics of the mammogram other than PD may be related to breast cancer. For example, Wolfe’s parenchymal patterns [5] indicate *texture* characteristics that are not necessarily correlated with PD [6]. Similarly, the American College of Radiology Breast Imaging Reporting and Data System (BI-RADS) classifies density into categories based on not only the amount of density but on descriptors of the distribution, such as “scattered” and “heterogeneously dense” [7]. This suggests that the pattern or texture of dense tissue should be considered while assessing mammograms. In addition, some texture features have been suggested to predict *BRCA1*/*2* carrier status, in contrast with PD [8].

A growing body of literature has considered mammographic texture features and their relationship with breast cancer risk. A recent review paper identified 17 original research articles [9]. These included early work by Manduca et al. [6], who identified texture features based on the grey-level co-occurrence matrix (GLCM) of neighbouring pixels. Kontos et al. [10] looked at a range of texture features with the aim to see how they are associated with PD based on both digital mammography and digital breast tomosynthesis (DBT). With a limited sample size, they identified GLCM features (homogeneity, contrast and energy) that were associated with PD from DBT and, to a lesser extent, digital mammography. Haberle et al. [11] considered five types of texture feature, finding that statistical features based on GLCM were strongly predictive of breast cancer, and that PD did not add information to risk once texture features had been accounted for. Li et al. [3] found that textural features predict breast cancer slightly better than semi-automated percent density. Keller et al. [12] compared risk prediction models using PD along with texture features based on the GLCM, statistical moments and run-length [13], and reported that texture features outperformed PD. However, there is not a great deal of consistency in the textural features identified between studies, so more work in this area is critically important. Nielsen et al. [14] developed a mammographic texture resemblance (MTR) marker based on multi-scale Gaussian features, which was found to have similar prediction performance compared with PD and could further improve the predictive ability when PD and MTR are combined.

While several studies have identified texture features for cancer prediction, many have been based on digitised film [9]. With the introduction of FFDM breast screening, there is a need to assess how best to assess the risk from textural features using digital mammograms. This is important partly because the properties of FFDM images differ from those of digitised films. For example, FFDM have a higher dynamic range than digitised film images [15] resulting in richer grey-level profiles; they also have different noise properties because the inherent granularity of screen-film mammography is not present in FFDM [16].

Very few studies have looked at texture features of original raw FFDM images. An additional issue with digital processed images is that one has to rely on manufacturers’ proprietary processing algorithms before feature extraction, which may result in images from different machines being less comparable. A recent review [9] of texture features for breast cancer risk found just two case-control studies based on raw FFDM including those by Chen et al. [17] and Zheng et al. [18]. There were just 156 cases in these studies (combined), and the case mammograms were from the contralateral (unaffected) breast at breast cancer diagnosis. Thus, overall information on the ability of textural features to predict risk of breast cancer in this context is limited.

The aim of this study was to develop a fully automated texture feature extraction system for raw digital mammograms, and to assess the predictive ability of textural features to stratify risk beyond volumetric PD. *Fully automated* in the context of this paper refers to a texture feature extraction system, including any pre-processing procedure, which operates without any human intervention.

## Methods

### Setting and study design

Two case-control studies were designed using women recruited to the Predicting Risk Of breast Cancer At Screening (PROCAS) cohort, in Manchester, UK [19]. The first case-control study was for feature selection (the training set), and cases were women with cancer detected at first screen on entry to PROCAS. Women were matched approximately 3:1 (controls vs cases) by age, body mass index (BMI), hormone replacement therapy (HRT) use and menopausal status. For feature selection the craniocaudal (CC) views of the contralateral breast for cases and the left breast for controls were used [20]. Unaffected breasts were followed up and recorded and cases of bilateral cancer were excluded. The average follow-up time was 3.9 years for cases and 4.9 years for controls.

The second case-control study was used to validate the risk score (the validation set). Each woman had a normal screening mammogram (no cancer detected) on entry to PROCAS, but an interval or screen-detected cancer had arisen subsequently. The mammograms were acquired approximately 3 years prior to diagnosis of breast cancer and were sampled independently from the same cohort as the training set. There is a small overlap of controls between the two datasets (n = 45) representing 2.7% of the total number of controls in both datasets. Again women were matched approximately 3:1 (controls vs cases) by age, BMI, HRT use, menopausal status and year of mammogram at entry. Since the validation was done in a double-blind fashion, case-control status was unknown before validation, so a pre-defined list of which breast was affected in each woman was provided so that the contralateral breast (also CC views) for cases and the same side for controls were used. As with the first study, women with bilateral cancer were excluded. The average follow-up time to date of diagnosis was 3.0 years for cases and the average follow-up time was 4.3 years for controls.

### Mammograms

All digital raw (“for processing”) mammograms were acquired using a GE Senographe system. The resolution of the mammograms was 10.628 pixels per mm. Percent volumetric density was assessed using Volpara 1.5.0 (Volpara Health Technologies, Wellington, New Zealand).

### Texture features

Texture features were extracted from the whole breast as a single region after windowing. Specifically, the minimum pixel value (whitest area) in the breast region was used as the lower bound of the window, and the value at the 75th percentile of the pixel value range (darker areas) within the breast was taken as the upper bound. The lower and upper bounds of the window were then reset (lower bound to 1 and pixels on or above the upper bounds to 0, which as a result also inverted the image) and the rest of the pixel values were linearly rescaled between 0 and 1.

We generally followed the literature to decide whether a feature was considered for evaluation in the training set. Statistical moments of pixel values from the windowed images were calculated directly in addition to features based on a grey-level co-occurrence matrix (GLCM), neighbourhood grey-tone difference matrix (NGTDM), form and shape of breast boundary, run-length, and grey-level size zone matrix (GLSZM) [3, 6, 10, 11, 13, 20–23].

Texture features were extracted from images at their original resolution. In addition, since some features (GLCM, NGTDM, run-length and GLSZM) are resolution-sensitive and might be associated with risk differently at different scales, they were extracted at reduced resolutions, by factors of 2, 4, 6, 8, 16, 32 and 64 using bi-cubic interpolation [6].

All texture features were calculated using Matlab (Mathworks, Natick, MA, USA). The Matlab package developed by Vallieres et al. [24] was employed for computing the GLCM, NGTDM, run-length and GLSZM features; and for these features, pixels were grouped equally into 10 grey levels in forming the relevant matrices before computing the texture features. A total number of 327 features were identified to be investigated. The full list of texture features considered and the types of features, downsize factors and univariate goodness-of-fit statistics using the training set are provided in the supplementary file (see Additional file 1).

### Statistical analysis

#### Feature selection and model building

An initial screening was performed to remove features that were correlated with any other feature with absolute Pearson correlation greater than 0.95, where the feature taken forward was randomly selected. This resulted in a total of 112 candidate texture features.

Feature selection was based on the least absolute shrinkage and selection operator (LASSO) method, adjusted for age, BMI and volumetric PD. The tuning parameter that controls the extent of coefficient shrinkage was chosen by cross-validation. The final calibrated model was based on the one standard error rule, where the most parsimonious model with error (deviance in this case) within one standard error of the model with minimum cross-validation error (leave one out) was selected [25]. LASSO feature selection was performed using the implementation by Friedman et al. [26] in the statistical software R [27]. A single risk score based on the LASSO fit was taken forward for validation. In addition, Volpara density grade (VDG), a categorical version of estimated volumetric PD, was also tested to see whether VDG added information to volumetric PD or selected texture features.

#### Validation of risk score and components

The composite risk score and individual texture features identified by LASSO were validated in a two-stage double-blind fashion. A statistical analysis plan was drafted detailing the procedure of data exchange and statistical analysis. After identification of a limited set of textural features and a risk score to investigate further using the training data, CW calculated these features using anonymised mammograms from the validation set, and blind to case-control status. EH ran the initial statistical analysis for these features using the validation set, and then CW was unblinded. The predictive ability of the risk score beyond volumetric PD was tested using conditional logistic regression. Deviance (or likelihood-ratio χ^2^) and the matched concordance index (mC) [28] were calculated to test and measure prediction performance. Deviance is a likelihood-based statistic and is analogous to the sum of squared residuals. For model comparison, it is common practice to examine the change in deviance (likelihood-ratio χ^2^) to measure relative model performance. mC is a modification of the concordance index (or area under the receiving operator characteristic curve (AUC)) to matched case-control studies, and measures an average concordance index within matched groups. Some other features that were not selected by LASSO but had previously been identified to be important, and were observed to be univariately significant in the training set (i.e. standard deviation, coarseness and contrast as shown below), were also assessed in the validation case-control study. As biologic phenotypes between screen-detected and interval cancers are different, the effects of texture features or volumetric PD on risk may also differ between them. To explore this, a series of multivariate models were fitted with risk factors that were statistically significant in the univariate models, and an additional interaction term between the image feature and indicator for screen-detected or interval cancer.

## Results

### Study characteristics

The training case-control study had a total of 264 cases and 787 controls, of which 199 cases were invasive tumors, 63 were ductal carcinoma in situ (DCIS), and two were unknown. The validation case-control study had a total of 317 cases and 931 controls, of which 277 were invasive tumors, 39 were DCIS and one was unknown. The demographic characteristics of the women in the two studies are summarised in Table 1, which shows that age, BMI, and HRT use were well-matched between cases and controls in both studies. As expected, median volumetric PD was greater in cases than controls in both studies. The median 10-year Tyrer-Cuzick score was also greater for cases than controls in both studies. A majority of women had never used HRT and the percentage was slightly higher in the training set (60% for controls and 65% for cases in training set, vs 51% for controls and 52% for cases in validation set; the differences between training and validation sets are significant with *p *values of 0.0002 and 0.0019, respectively). In both studies around three quarters of women were postmenopausal, and the majority of women were ethnically white.

**Table 1:** Demographics of the training set (cancers detected at first screen on entry to the PROCAS study) and validation set (cancers detected at a subsequent screen or between screening rounds)

	Training set					Validation set				
	Controls		Cases		*P* value	Controls		Cases		*P* value
	N (%)		N (%)			N (%)		N (%)		
Age at consent (years)					0.9994					0.9997
<50	44	(6)	16	(6)		46	(5)	16	(5)	
50–54	200	(25)	65	(25)		193	(21)	64	(20)	
55–-59	150	(19)	51	(19)		164	(18)	58	(18)	
60–64	172	(22)	57	(22)		286	(31)	96	(30)	
65–69	166	(21)	57	(22)		195	(21)	67	(21)	
70+	55	(7)	18	(7)		47	(5)	16	(5)	
HRT use					0.2234					0.9646
Unknown	11	(1)	7	(3)		22	(2)	6	(2)	
Never	473	(60)	171	(65)		475	(51)	165	(52)	
Previous	262	(33)	72	(27)		329	(35)	110	(35)	
Current	41	(5)	14	(5)		105	(11)	36	(11)	
BMI (kg/m^2^)					0.9797					0.9408
Unknown	-		-			-		1	(0)	
<25	241	(31)	80	(30)		335	(36)	117	(37)	
25–29	289	(37)	96	(36)		341	(37)	113	(36)	
≥30	257	(33)	88	(33)		255	(27)	86	(27)	
Menopausal status					0.9914					0.9887
Unknown	16	(2)	7	(3)		32	(3)	12	(4)	
Perimenopausal	94	(12)	32	(12)		134	(14)	46	(15)	
Postmenopausal	591	(75)	196	(74)		698	(75)	237	(75)	
Premenopausal	86	(11)	29	(11)		67	(7)	22	(7)	
Ethnic origin					0.0411					0.2229
Other/unknown	38	(5)	22	(8)		81	(9)	35	(11)	
White	749	(95)	242	(92)		850	(91)	282	(89)	
Parity					0.8143					0.0351
Unknown	-		-			1	(0)	4	(1)	
Nulliparous	97	(12)	34	(13)		90	(10)	44	(14)	
Parous	690	(88)	230	(87)		840	(90)	269	(85)	
Tyrer-Cuzick (10-year risk, %) (median, Q1–Q3)	2.73	(2.19–3.60)	2.82	(2.29–3.88)	0.0028	2.68	(2.09–3.55)	2.91	(2.24–4.03)	<0.0001
Volumetric PD (median, Q1–Q3)	5.34	(4.06–7.35)	5.88	(4.62–8.55)	0.0003	4.73	(3.50–6.92)	5.31	(3.79–7.57)	0.0041

*P* values, from likelihood-ratio chi-square tests, indicate whether there are significant difference between cases and controls

*HRT* hormone replacement therapy, *BMI* body mass index, *Q1* 25th percentile, *Q3* 75th percentile, *PD* percent density

### Texture feature risk score development

Three features were selected from the training set using LASSO (the value of the LASSO tuning parameter = 0.0402) and taken forward for validation in a combined risk score. They were the GLCM feature *sum average* calculated using images downsized by factors of 16, 32 and 64. Sum average is a feature considered to capture a relationship between radiolucent and radiopaque areas in an image [29]. Table 2 shows the correlation coefficients between the three sum average features, volumetric PD, age, BMI, and other important features identified in the literature including standard deviation (SD), contrast (based on NGTDM), and coarseness calculated at the original image resolution. Coarseness measures the amount of local grey-level variation and contrast measures the amount of difference among all grey levels and the amount of local variation in grey level presented in the image [21]. SD is a histogram-based feature so does not take into account spatial relationships between pixels.

**Table 2:** Spearman correlation coefficients between age, BMI, PD and texture features

	Age	BMI	Volumetric PD	Sum average 16	Sum average 32	Sum average 64	SD	Coarseness	Contrast
Age	1								
BMI	0.03	1							
Volumetric PD	-0.14	-0.57	1						
Sum average 16	-0.19	-0.35	0.63	1					
Sum average 32	-0.23	-0.33	0.63	0.81	1				
Sum average 64	-0.18	-0.23	0.54	0.74	0.88	1			
SD	-0.16	-0.19	0.46	0.27	0.32	0.31	1		
Coarseness	-0.12	-0.62	0.79	0.49	0.58	0.51	0.53	1	
Contrast	0.15	0.34	-0.74	-0.45	-0.52	-0.52	-0.64	-0.80	1

Sum average 16, 32 and 64 are texture feature sum average using images downsized by a factor of 16, 32 and 64

*PD* percent density, *BMI* body mass index, *SD* standard deviation

The sum average features at different resolutions were relatively highly and positively correlated (Spearman correlation 0.74–0.88). There were weaker and negative associations between sum average features and age (-0.23 to -0.18) or BMI (-0.35 to -0.23). Volumetric PD was quite strongly and positively correlated with the sum average features (0.54– 0.63).

Table 3 shows the prediction performance of volumetric PD and the three sum average features in a univariate analysis, in addition to some texture features that have previously been identified in the literature and were univariately significant in the training data, and taken forward to be assessed in the validation set as secondary measures.

**Table 3:** Univariate modelling results from the training dataset

Parameter	Standardized odds ratio	95% CI for odds ratio	χ^2^	*P* value	mC	95% CI for mC
Coarseness	1.22	(1.06–1.41)	7.28	6.98E-03	0.58	(0.53– 0.62)
Contrast	0.73	(0.62–0.85)	16.75	4.27E-05	0.40	(0.36– 0.45)
SD	1.32	(1.13–1.54)	13.11	2.94E-04	0.57	(0.52– 0.61)
Sum average 16	1.52	(1.31–1.77)	31.26	2.25E-08	0.61	(0.56– 0.65)
Sum average 32	1.52	(1.31–1.77)	31.75	1.75E-08	0.61	(0.56– 0.66)
Sum average 64	1.48	(1.28–1.71)	29.07	6.98E-08	0.58	(0.53– 0.63)
Volumetric PD	1.36	(1.18–1.57)	18.05	2.16E-05	0.59	(0.55– 0.64)

Total number of observations (*N*) = 1051, including 264 cases and 787 controls. Standardized odds ratio is the change in odds for a standard deviation (in controls) increase in predictors, adjusted for age and body mass index. Sum average 16, 32 and 64 are the texture feature sum average using images downsized by a factor of 16, 32 and 64

*PD* percent density, *CI* confidence interval, *mC* matched concordance index, *SD* standard deviation

In the training sample, all three sum average features outperformed the other univariate features in terms of χ^2^, and achieved a mC that was comparable with PD. Sum average downsized by a factor of 32 achieved the best result in terms of both χ^2^ and mC (0.61). The performance of PD, SD and contrast was similar, while coarseness was the least predictive in terms of χ^2^. We also tested the Volpara density grade (VDG), a categorical version of estimated volumetric PD, finding it has a very similar predictive performance compared to volumetric PD (χ^2^ = 20.19, degrees of freedom = 3). A series of likelihood-ratio tests showed that VDG did not add further information to either volumetric PD (Δχ^2^ = 3.36, *p* = 0.3), or LASSO selected texture features such as sum average 16 (Δχ^2^ = 3.40, *p* = 0.3).

The risk score taken forward for validation is a weighted linear combination of the three sum average features. The standardized weights (i.e. using *z* scores where predictors were rescaled by their means and standard deviations before entering the model) were:

$$ \mathrm{Risk}\kern0.17em \mathrm{score}={0.044}^{\ast}\mathrm{SumAverage}16+{0.036}^{\ast}\mathrm{SumAverage}32+{0.066}^{\ast}\mathrm{SumAverage}64 $$

where the means of the three features were respectively 0.0555, 0.0559 and 0.0566; the standard deviations were respectively 0.000238, 0.000430 and 0.000775. It can be seen that sum average 64 contributed most to the score (0.066/(0.066 + 0.036 + 0.044) = 45%). In the training set the risk score had a similar mC (0.60) to its sum average components.

**Fig. 1:**
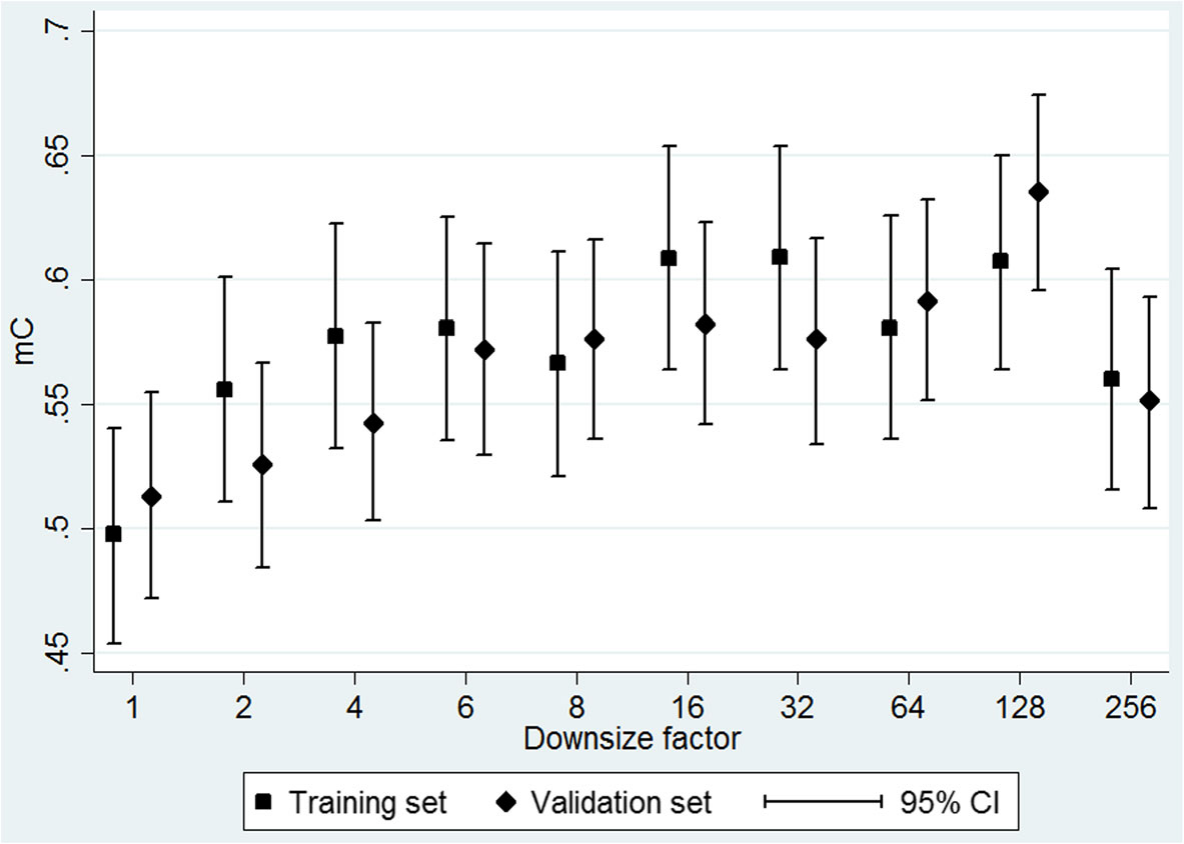
Matched concordance index (mC) for sum average at different image downsize factors, with bootstrap 95% confidence intervals (CI)

Figure 1 shows the mC and its confidence intervals for the sum average features calculated at different resolutions, including those not selected by the LASSO algorithm. Generally mC increased as images were downsized up to a factor of 32, and was approximately flat at downsizing factors between 16 and 128.

**Fig. 2:**
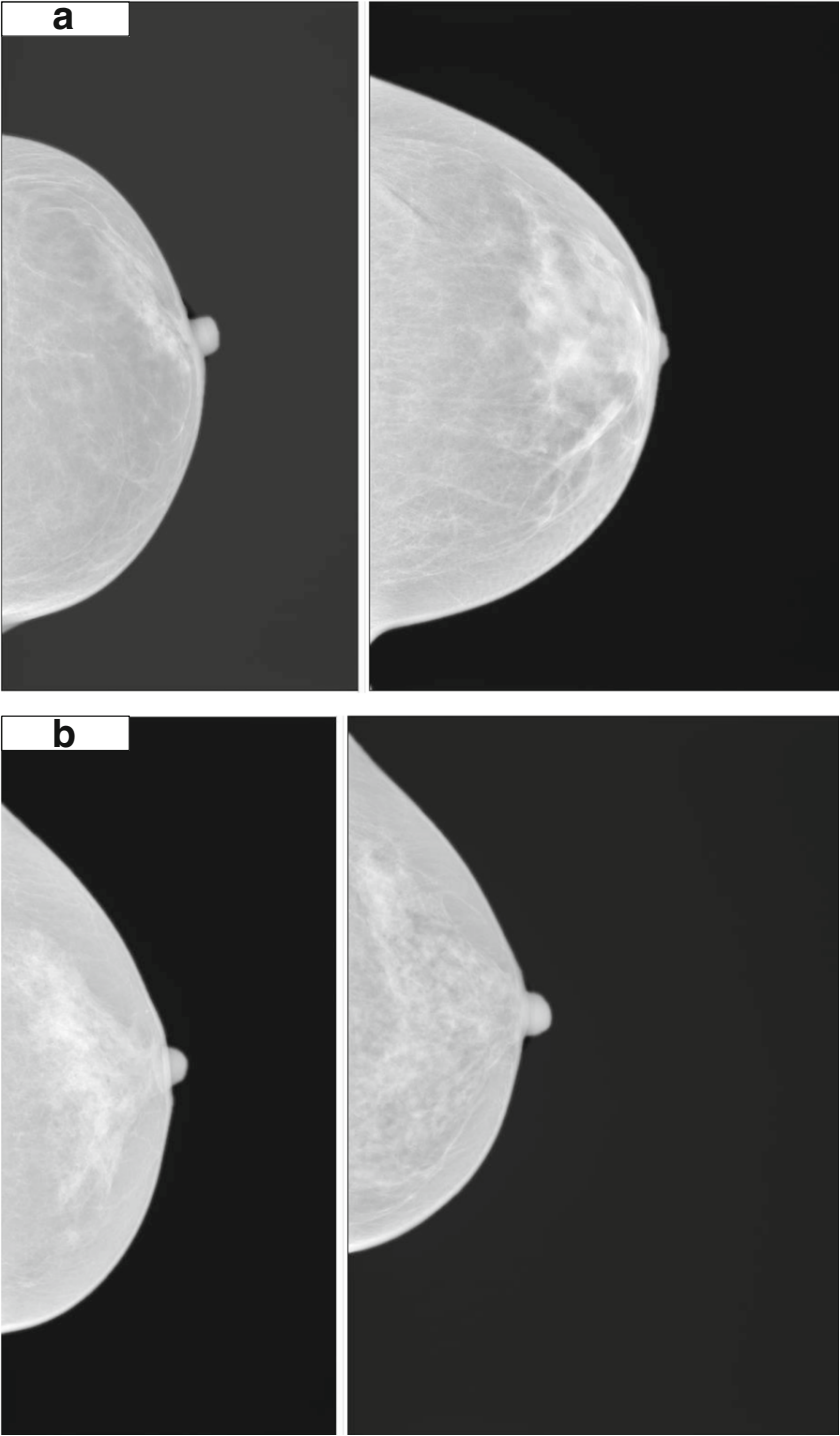
Comparison of mammograms (for presentation purpose processed images are shown) with two of the lowest (**a**) and highest (**b**) standardized risk scores. All mammograms have similar volumetric percent density (PD) around 10%. **a** Mammograms with low risk scores (-1.7 and -1.3, respectively). Volumetric PDs are 10.1% and 10.2%, respectively. **b** Mammograms with high risk scores (3.2 and 2.0, respectively). Volumetric PDs are 9.9% and 10.0%, respectively

To better understand the feature sum average and risk score, and see how the feature looks visually, example images with low and high values of risk scores but similar volumetric PDs in the training study are presented in Fig. 2.

### Validation of texture risk score

The regression results using the validation dataset in Table 4 confirmed the predictive power of the texture risk score found in the training dataset. The standardized odds ratio was 1.36 (95% CI 1.20–1.55) with mC 0.58 (95% CI 0.54–0.62), which was broadly comparable with the development analysis using the training set (mC = 0.60). The risk score also achieved a better performance than volumetric PD in terms of deviance (Δχ^2^ = 10.55), indicating some evidence of preference of risk score relative to the PD [30] (logarithm PD Δχ^2^ = 6.95), but a similar mC (0.58 compared with 0.57 for PD). A series of likelihood-ratio tests showed that the risk score also added independent predictive information to volumetric PD (Δχ^2^ = 14.38, *p* = 0.0008) and Tyrer-Cuzick risk (logarithm transformed, Δχ^2^ = 22.43, *p* < 0.0001) and PD and Tyrer-Cuzick combined (Δχ^2^ = 10.22, *p* = 0.001). On the other hand, once the risk score was taken into account, PD added little information (Δχ^2^ = 0.21, *p* = 0.7).

**Table 4:** Univariate modelling results using the validation dataset

Parameter	Standardized odds ratio	95% CI for odds ratio	χ^2^	*P* value	mC	95% CI for mC
Risk score	1.36	(1.20–1.55)	22.39	2.22E-06	0.58	(0.54– 0.62)
Coarseness	1.06	(0.92–1.22)	0.61	4.34E-01	0.50	(0.46– 0.54)
Contrast	0.87	(0.76–0.99)	4.33	3.75E-02	0.46	(0.42– 0.50)
SD	1.01	(0.89–1.15)	0.03	8.55E-01	0.50	(0.45– 0.54)
Sum average 16	1.29	(1.14–1.47)	15.37	8.85E-05	0.58	(0.54– 0.62)
Sum average 32	1.32	(1.1–1.50)	17.55	2.80E-05	0.58	(0.53– 0.62)
Sum average 64	1.38	(1.21–1.57)	23.81	1.06E-06	0.59	(0.55–0.63)
Volumetric PD	1.27	(1.11–1.46)	11.84	5.80E-04	0.57	(0.53– 0.61)

Total number of observations (N) = 1248, including 317 cases and 931 controls. Standardized odds ratio is the change in odds for a standard deviation (in controls) increase in predictors, adjusted for age and body mass index. Sum average 16, 32 and 64 are the texture feature sum average using images downsized by a factor of 16, 32 and 64.

*PD* percent density, *CI* confidence interval, *mC* matched concordance index, *SD* standard deviation

Looking at individual texture features, only the three sum average features and contrast were statistically significant. Sum average based features also achieved the best fit in terms of deviance compared with other texture features and PD. Additionally, only the sum-average-based features added information to PD (the Δχ^2^ test statistics were 5.16, 6.46 and 12.56 for sum average using images downsized by factors of 16, 32 and 64, respectively). This confirms that sum average at low resolutions is an independent risk factor. Other texture features did not add further information once the risk score was taken into account.

Modelling results showing the difference between screen-detected and interval cancers for statistically significant features are presented in Table 5. As Table 5 shows, with the exception of contrast, the difference in screen-detected and interval cancers was statistically significant; and texture features and volumetric PD had higher odds ratios for interval than screen-detected cancers. This is likely related to masking of interval cancers from dense breasts, and perhaps also masking due to texture when the density is more dispersed (higher sum average value, c.f. Fig. 2).

**Table 5:** Modelling results for screen-detected and interval cancer

	Risk score	Contrast	Sum average 16	Sum average 32	Sum average 64	Volumetric PD
Standardized OR for screen-detected cancer	1.15	0.88	1.09	1.11	1.20	1.13
	(0.99–1.35)	(0.75–1.04)	(0.93–1.26)	(0.95–1.30)	(1.02–1.40)	(0.94–1.35)
Standardized OR for interval cancer	2.09	0.84	2.12	2.15	1.91	1.53
	(1.59–2.74)	(0.66–1.06)	(1.59–2.81)	(1.62–2.86)	(1.48–2.47)	(1.21–1.92)
Δχ^2^	15.27	0.15	18.53	17.84	9.80	4.38
*P* value	0.0001	0.70	<0.0001	<0.0001	0.002	0.036

Standardized odds ratio (OR) is the change in odds for a standard deviation (in controls) increase in image features; the 95% confidence intervals are in brackets. Sum average 16, 32 and 64 are the texture feature sum average using images downsized by a factor of 16, 32 and 64

The Δ*χ* ^2^ and *p* values refer to likelihood-ratio tests on whether there is a significant difference between screen-detected and interval cancers (i.e. significance of interaction terms)

*PD* percent density

## Discussion

This study aimed to predict breast cancer risk with various texture features from raw digital mammograms. To achieve this, a number of relevant texture features were extracted and the LASSO model was employed for feature selection. The risk score was validated using a separate set of cases and controls from the same overall cohort.

The original raw mammogram files were pre-processed using a windowing technique. This effectively means that the darkest 25% pixels within the breast (mostly the uncompressed region) were set to be background. This is similar to the method used by Heine et al. [20] for computing standard deviation. They eroded a 25% area from the edge of the breast in scanned film images, as they reported that the region in question could potentially interfere with further feature extraction. The breast edge contains the darkest pixels. In addition to standardizing pixel intensities, another benefit of windowing is that image contrast is enhanced, making image appearance similar to that of film mammograms.

The texture features tested included many of those identified in previous studies, such as standard deviation of the pixel intensity values, NGTDM contrast, coarseness, and GLCM features. We also assessed some novel features that have been less well-studied in the literature, including GLSZM-based features that measure zonal effects and some form-based features such as the diameter of a circle with the same area as the breast region.

The GLCM feature, sum average, at lower image resolutions was selected by LASSO in the training study. Based on its mathematical formulation (see Appendix) and visual assessment of some mammograms, one can show that this feature tends to identify dispersed patterns of density on a mammogram. It was slightly surprising that PD and some previously reported texture features such as standard deviation, contrast and coarseness were not selected, although contrast was significantly and negatively associated with risk in both the training and validation studies, in line with Huo et al. [21]. Other texture features such as standard deviation and coarseness, however, were not significant in the validation study. While it is interesting that only 3 features were selected out of 112 features by the LASSO algorithm, it is worth noting that the features that were not selected by LASSO are not necessarily non-predictive of risk. For example, the feature contrast was shown as predictive in both the training and validation studies. Volumetric PD was not selected by LASSO either. This may be an indication that once some features were used, other features may no longer have added information. This is supported by the likelihood-ratio test that showed that once the risk score is taken into account, volumetric PD adds little information (*p* = 0.7). In the validation study, the three sum-average-based features achieved the best results among univariate predictors in terms of both deviance and mC. The risk score, a weighted combination of three sum-average-based features, has only obtained similar deviance or mC to its components on univariate analysis, suggesting sum average measured at one image resolution might be adequate. Although the sum average feature has been employed in some previous studies, it has not previously been identified as the strongest texture feature. The reason for different findings might be due to differences in the methods used to compute textural features. Indeed, it is often difficult to determine precisely how a feature was computed in prior publications and so we have been careful to provide a detailed description of the sum average feature used here in the Appendix. A lesser factor for differences might be different feature selection methods. Previous studies have often used stepwise regression for feature selection [6, 8, 11, 12, 21]. However, as pointed out by Hastie et al. [25], stepwise regression often leads to poor results compared to a less greedy method such as LASSO.

We explored the risk score by visual inspection of mammograms. Those in Fig. 2 are deliberately extreme, but they were chosen to show readers a clear demonstration that mammograms with a high risk score have more dispersed areas of bright pixels; whilst those with a low risk score do not. The example shows that a higher risk score helps to identify more widely dispersed dense patterns. In other words, it might capture an element of dense area that is (implicitly) not necessarily taken into account by volumetric density. As observed in Table 2, there is fairly high correlation between texture features and PD, so some of the effects of PD may be captured by texture features. Considering texture features improve prediction beyond PD, it is possible some spatial patterns of dense tissue may be related to risk in addition to the relative amount of density. This interpretation also follows the mathematical formula for the feature. Downsizing is important because it enables the measure of spatial relationships between pixels at a greater distance, and so better measures wider areas of density. In summary, this feature seems to capture the distribution of dense tissue and our results suggest that mammograms with greater areas of high density are associated with higher risk.

Differences in prediction performance at different resolutions are due to change in patterns for each feature at those resolutions. Some texture features are more consistent than others when the images were re-scaled. For example, the Spearman correlation coefficient for sum average between downsize factors of 1 and 64 is -0.12, indicating weak association; whilst the Spearman correlation coefficient for coarseness between downsize factors of 1 and 64 is 0.78, showing strong correlation. This means some factors such as coarseness are more consistent than others when the images were re-scaled. Texture features such as those based on GLCM typically measure spatial relationships between a pixel and its neighbouring pixels. As the images were downsized, the neighbouring pixels become more distant, thus resulted in changes in feature patterns. Some features, such as coarseness, are relatively robust to such change in neighbourhood definition, while some features change dramatically. This suggests that it is important to consider the impact of image resolution while analysing a certain texture feature. The implication is that a feature that predicts well at a given resolution may not perform well at another resolution. It is thus important to indicate the image resolution when exploring the prediction performance of a feature. This finding has also been observed elsewhere. For example, Haberle et al. [11] reported that a GLCM feature based on the same set of mammograms but at different resolutions have either different (opposite) associations with PD or different associations with cancer risk. Manduca et al. [6] also found that texture features tended to predict risk better when they were extracted at reduced image resolutions. For instance, the AUC of a feature increased from 0.50 to 0.60 when the images were downsized by factors of 2, 4, 8, and 16.

One contribution of our paper is that it shows how to extract a useful textural feature in a fully automated way from digital raw mammograms. Traditionally, studies utilising image texture features for cancer prediction have been based on scanned films, e.g. [6, 21]. Also, there is concern that results from processed (i.e. for presentation) mammograms may not be generalizable since different manufacturers have their own proprietary processing algorithms, making the resulting images and their features potentially not fully comparable between different manufacturers and machines. This paper addresses the above concerns by using the raw FFDM, and has shown which texture features might be important for predicting breast cancer risk, and how the risk model can be improved by downsizing the images. It is anticipated that the method proposed in the paper would better facilitate breast cancer risk prediction by using digital mammograms.

There are several possible ways to expand our study. For example, our image pre-processing method did not consider acquisition parameters, such as compression force, and thickness of the compressed breast and breast edge. It is possible that employing these acquisition parameters may lead to better image pre-processing and ultimately risk prediction. Another very important direction is to externally validate the method on a different population with different characteristics such as ethnicity and parity. In particular, we note that more than 92% of our study population was white, and more than 88% parous. The use of larger and diverse datasets would allow for additional breast cancer risk factors to be adjusted in the model. Also, the mammograms used in our analysis were all acquired from a GE system. It would be interesting to test our method on mammograms produced by other brands of machines. For transferability of our method, digital mammograms from other machines may be re-scaled to the same resolution as in this paper before feature extraction. There is also potential that our method can be adapted for digitised films. It would also be interesting to compare our method to recent advancement in deep learning [31]. Finally, this study focused on the CC view of mammograms. It is possible that texture features that are predictive of cancer risk may be different for mediolateral oblique (MLO) view mammograms. The issue with using MLO view mammograms is how to treat the pectoral muscle in an analysis. One possible approach is to remove the pectoral muscle before feature extraction. This requires an automated pectoral muscle removal algorithm (e.g. [17]) since our ultimate aim is to develop a fully automated risk prediction system. The additional information from the MLO view may assist in better predicting breast cancer risk than using the CC view alone.

## Conclusion

This paper has shown that texture features are likely to be useful for predicting breast cancer risk using raw digital mammograms. Important texture features previously identified in the literature and some novel features were tested. The feature selection method LASSO was adopted to finalise the feature set taken forward for validation.

Among various features tested including standard deviation, coarseness, contrast and volumetric PD, we found the GLCM feature sum average at low image resolution was the strongest predictor of breast cancer risk, and added independent information to volumetric PD. An image standardization method was adopted to pre-process the digital raw mammograms before feature extraction, making it likely that our approach would have merit on other mammogram machines. However, while the selected features and calibrated model were internally validated in a separate case-control study from the same cohort with consistent results, our findings and risk algorithm would benefit from further studies to externally validate them.

### Additional files

Additional file 1: Univariate modelling results from training dataset. This table shows the univariate modelling results of all candidate texture features considered using training dataset. (DOCX 76 kb)

## Appendix

Sum average is a statistical texture feature computed from grey-level co-occurrence matrix (GLCM) constructed by considering how often pairs of pixels with specific values and in a specified spatial relationship occur in an image. Sum average is defined as [32]:

$$ \left[{\sum}_{i,j}\left(i+j\right)\cdot p\left(i,j\right)\right]/\left(2{I}^2\right) $$

where *I* is the total number of grey levels, and *p*(*i*, *j*) denotes the proportion of a pixel at grey level *i* in a defined spatial relationship with a pixel at grey level *j* in the image. *p*(*i, j*) is obtained by firstly counting the frequencies of pairs of pixels at different grey levels with a defined spatial relationship (in this study, all eight directions surrounding a pixel were counted), and then normalized so that the sum of the elements of the GLCM is equal to 1. The general forumula includes *I* ^2^ to make it comparable between different sizes of GLCMs. In this study, the number of grey levels of 10 was adopted (i.e. *I* = 10), and the pixels within the breast region was divided into 10 levels in such a way that each level has equal probability. We tested using the number of grey levels other than 10 and the feature pattern changed only marginally - for instance using 5 grey levels the correlation coefficient with sum average using 10 grey levels was 0.97.

Sum average can be seen as the weighted (by grey levels) sum of GLCM elements. Thus this texture is likely to have higher value if many high grey-level pixels are close to each other.

In addition to sum average, the following GLCM features were tested:

Contrast:

$$ {\sum}_{i,j}{\left|i-j\right|}^2p\left(i,j\right) $$

Correlation:

$$ {\sum}_{i,j}\frac{\left(i-{\mu}_i\right)\left(j-{\mu}_j\right)p\left(i,j\right)}{\sigma_i{\sigma}_j} $$

where μ σ are means and standard deviations of corresponding rows and columns of GLCM.

Dissimilarity:

$$ {\sum}_{i,j}\mid i-j\mid p\left(i,j\right) $$

Energy:

$$ {\sum}_{i,j}p{\left(i,j\right)}^2 $$

Entropy:

$$ {\sum}_{i,j}-p\left(i,j\right)\log \left(p\left(i,j\right)\right) $$

Homogeneity:

$$ {\sum}_{i,j}\frac{p\left(i,j\right)}{1+\left|i-j\right|} $$

Variance:

$$ \left[{\sum}_{i,j}\left({\left(i-{\mu}_i\right)}^2+{\left(j-{\mu}_j\right)}^2\right)\cdot p\left(i,j\right)\right]/\left(2{I}^2\right) $$

Similar to GLCM, a *neighbourhood grey-tone difference matrix* (NGTDM) could be constructed, and relevant texture features could be extracted by computing the summary statistics of NGTDM. The NGTDM is a vector (column matrix) constructed by firstly calculating the average grey tone over a neighbourhood centred at, but excluding (k,l):

$$ {\overline{A}}_l=\overline{A}\left(k,l\right)=\frac{1}{W-1}\left[\sum_{m=-d}^d\sum_{n=-d}^df\left(k+m,l+n\right)\right] $$

where (*m*, *n*) ≠ (0, 0) (i.e. excluding the (*k,l*)); f(*k,l*) is the grey tone of any pixel at (*k,l*) having grey tone value *i*; *d* specifies the neighbourhood size (d = 1 in this case); and *W* = (2*d* + 1)^2^. Then the *i*th element of the NGTDM is:

$$ s(i)=\left\{\begin{array}{c}\sum \left|i-{\overline{A}}_l\right|,\kern1.08em for\;i\in {N}_i, if\;{N}_i\ne 0\\ {}0,\kern2.04em otherwise\end{array}\right. $$

where *N*_*i*_ is the set of all pixels having grey tone *i* (exluding the peripheral regions of width *d*).

Coarseness is defined as:

$$ {\left[\upepsilon +{\sum}_i{p}_is(i)\right]}^{-1} $$

where ε is a small number (2^-52^ in this case) to prevent it becoming infinite; *p* is the probability of occurrence of the corresponding intensity value. Contrast is defined as:

$$ \left[\frac{1}{N_g\left({N}_g-1\right)}{\sum}_i{\sum}_j{p}_i{p}_j{\left(i-j\right)}^2\right]\left[\frac{1}{n^2}{\sum}_is(i)\right] $$

where *N*_*g*_ is the total number of different grey levels in the image; n = N-2d.

Coarseness and contrast have been successfully applied for classification of cancer or BRCA1/2 status in the literature, such as Huo et al. [21] and Kontos, et al [10] where these features were described. As with GLCM, the pixels within the breast region were equally divided into 10 levels. In addition to coarseness and contrast, the following NGTDM features were tested:

Busyness:

$$ \left[{\sum}_i{p}_is(i)\right]/\left[{\sum}_{i,j}i{p}_i-j{p}_j\right],\kern0.37em {p}_i\ne 0,{p}_j\ne 0 $$

Complexity:

$$ {\sum}_{i,j}\left\{\left|i-j\right|/\left({n}^2\left({p}_i+{p}_j\right)\right)\right\}\left\{{p}_is(i)+{p}_js(j)\right\},{p}_i\ne 0,{p}_j\ne 0 $$

Strength:

$$ \left[{\sum}_{i,j}\left({p}_i+{p}_j\right){\left(i-j\right)}^2\right]/\left[\epsilon +{\sum}_is(i)\right],\kern0.37em {p}_i\ne 0,{p}_j\ne 0 $$

Similar to GLCM and NGTDM, run-length features were extracted from the run-length matrix. Let *p*(*i*, *j*) be the number of runs with pixels of grey level *i* and run-length *j*, *n*_*r*_ be the total number of runs, and *n*_*p*_ be the number of pixels in the region of interest:

Short run emphasis (SRE):

$$ \frac{1}{n_r}{\sum}_i{\sum}_j\frac{p\left(i,j\right)}{j^2} $$

Long run emphasis (LRE):

$$ \frac{1}{n_r}{\sum}_i{\sum}_jp\left(i,j\right)\cdot {j}^2 $$

Grey-level non-uniformity (GLN):

$$ \frac{1}{n_r}{\sum}_i{\left({\sum}_jp\left(i,j\right)\right)}^2 $$

Run length non-uniformity (RLN):

$$ \frac{1}{n_r}{\sum}_j{\left({\sum}_ip\left(i,j\right)\right)}^2 $$

Run percentage (RP):

*n*_*r*_/*n*_*p*_

Low grey-level run emphasis (LGRE):

$$ \frac{1}{n_r}{\sum}_i{\sum}_j\frac{p\left(i,j\right)}{i^2} $$

High grey-level run emphasis (HGRE):

$$ \frac{1}{n_r}{\sum}_i{\sum}_jp\left(i,j\right)\cdot {i}^2 $$

Short run low grey-level emphasis (SRLGE):

$$ \frac{1}{n_r}{\sum}_i{\sum}_j\frac{p\left(i,j\right)}{i^2\cdot {j}^2} $$

Short run high grey-level emphasis (SRHGE):

$$ \frac{1}{n_r}{\sum}_i{\sum}_j\frac{p\left(i,j\right)\cdot {i}^2}{j^2} $$

Long run low grey-level emphasis (LRLGE):

$$ \frac{1}{n_r}{\sum}_i{\sum}_j\frac{p\left(i,j\right)\cdot {j}^2}{i^2} $$

Long run high grey-level emphasis (LRHGE):

$$ \frac{1}{n_r}{\sum}_i{\sum}_jp\left(i,j\right)\cdot {i}^2\cdot {j}^2 $$

Grey-level variance (GLV):

$$ \sqrt{\frac{1}{I\cdot J}{\sum}_i{\sum}_j{\left[p\left(i,j\right)\cdot i-\mu \right]}^2} $$

where $$ \mu =\frac{1}{I\cdot J}{\sum}_i{\sum}_jp\left(i,j\right)\cdot i $$.

Run-length variance (RLV):

$$ \sqrt{\frac{1}{I\cdot J}{\sum}_i{\sum}_j{\left[p\left(i,j\right)\cdot j-\mu \right]}^2} $$

where $$ \mu =\frac{1}{I\cdot J}{\sum}_i{\sum}_jp\left(i,j\right)\cdot j $$.

The GLSZM features are similar to run-length features but the focus is on sizes of zones instead of collinear pixels (i.e. runs). In GLSZM, *p*(*i*, *j*) is defined as the number of zones with pixels of grey level *i* and area *j*, and the same formulas for run-length features can be used to compute GLSZM features.

As for shape-based features, convex area measures the number of pixels in the smallest convex polygon that contains the breast; equivalent diameter measures the diameter of a circle with the same area as the breast; extent measures the ratio of pixels in the breast to pixels in the total bounding box; major axis length is the length of the longest diameter of an ellipse that has the same normalized second central moments as the breast region; similarly minor axis length is the length of the minor axis of an ellipse that has the same normalized second central moments as the breast; and solidity is the ratio of breast area and its convex area.

The software implementing the method described in this paper has been made available for the Windows operating system (https://doi.org/10.6084/m9.figshare.4994429.v2). Upon launching the software, a dialogue box would be prompted asking users which mammogram file(s) to examine and where the results are to be saved. The software would then compute the texture features without further user input and save the results in a spreadsheet at the location the user specified.

Differences from density assessment case-control studies: the number of cases and controls differs from a report (submitted elsewhere) that compared density methods using the same women. The reasons are as follows. First, the training case-control study was a subset of one with 317 cases and 952 controls. Three women were excluded due to linkage errors between mammograms and questionnaire data (although they could be subsequently incorporated we decided to present the training data as it was undertaken before validation). We also excluded women with unknown BMI and volumetric PD at the time of analysis (79 and 37 women with missing BMI and PD, respectively). An additional 100 controls were removed during conditional logistic regression because they did not have matched cases as a result of the aforementioned exclusions.

The validation case-control study originally had 338 cases and 1014 controls: 23 women were excluded because of unavailability of mammograms at the time of validation (either no mammograms provided for some women at the given side; or only MLO views were available but no CC views). There were 64 further women removed because the side of cancer (left or right) was unknown; two women were further excluded due to lack of volumetric PD data at the time of validation. A further 15 controls were removed during conditional logistic regression because they had no matched cases as a result of the aforementioned exclusions.

## Abbreviations

AUC: Area under the receiver operating characteristic curve
BI-RADS: Breast Imaging Reporting and Data System
BMI: Body mass index
CC: Craniocaudal
CI: Confidence interval
DBT: Digital breast tomosynthesis
DCIS: Ductal carcinoma in situ
GLCM: Grey-level co-occurrence matrix
GLSZM: Grey-level size zone matrix
HRT: Hormone replacement therapy
LASSO: Least absolute shrinkage and selection operator
mC: Matched concordance index
MLO: Mediolateral oblique
MTR: Mammographic texture resemblance
NGTDM: Neighbourhood grey-tone difference matrix
OR: Odds ratio
PD: Percent density
PROCAS: Predicting Risk Of breast Cancer At Screening
SD: Standard deviation
VDG: Volpara density grade
