# Supplementary material for: A novel and fully automated mammographic texture analysis for risk prediction: results from two case-control studies
Source: Breast Cancer Res. 2017 Oct 18;19:114. doi: 10.1186/s13058-017-0906-6 (PMC5648465; doi:10.1186/s13058-017-0906-6)
Supplement: Supplementary file 1 — Univariate modelling results from training dataset. This table shows the univariate modelling results of all candidate texture features considered using training dataset. (DOCX 76 kb) [file 13058_2017_906_MOESM1_ESM.docx]

Additional file 1

Table S1 Univariate modelling results from training dataset

|  | | *Standardized Odds Ratio* | *95% CI for Odds Ratio* | | *χ^2^* | *p-value* | *mC* | *95% CI for mC* | | |
| --- | --- | --- | --- | --- | --- | --- | --- | --- | --- | --- |
| *Feature* | *Downsize* | 0.73 | (0.61 - | 0.88) | 11.42 | 7.26E-04 | 0.42 | (0.38 - | 0.47) |  |
| *Busyness* | *1* |  |  |  |  |  |  |  |  |  |
|  | *2* | 0.76 | (0.64 - | 0.91) | 9.04 | 2.65E-03 | 0.44 | (0.40 - | 0.49) |  |
|  | *4* | 0.82 | (0.69 - | 0.97) | 5.37 | 2.05E-02 | 0.46 | (0.42 - | 0.51) |  |
|  | *6* | 0.83 | (0.70 - | 0.99) | 4.56 | 3.26E-02 | 0.47 | (0.42 - | 0.51) |  |
|  | *8* | 0.85 | (0.71 - | 1.01) | 3.56 | 5.91E-02 | 0.47 | (0.43 - | 0.52) |  |
|  | *16* | 0.88 | (0.74 - | 1.04) | 2.15 | 1.43E-01 | 0.49 | (0.44 - | 0.53) |  |
|  | *32* | 0.92 | (0.78 - | 1.09) | 0.99 | 3.20E-01 | 0.49 | (0.45 - | 0.54) |  |
|  | *64* | 0.97 | (0.83 - | 1.14) | 0.13 | 7.17E-01 | 0.50 | (0.45 - | 0.54) |  |
| *Coarseness* | *1* | 1.22 | (1.06 - | 1.41) | 7.28 | 6.98E-03 | 0.58 | (0.53 - | 0.62) |  |
|  | *2* | 1.19 | (1.03 - | 1.38) | 5.52 | 1.88E-02 | 0.56 | (0.51 - | 0.60) |  |
|  | *4* | 1.15 | (0.99 - | 1.34) | 3.49 | 6.18E-02 | 0.54 | (0.49 - | 0.58) |  |
|  | *6* | 1.14 | (0.98 - | 1.33) | 2.87 | 9.05E-02 | 0.53 | (0.49 - | 0.58) |  |
|  | *8* | 1.13 | (0.97 - | 1.31) | 2.26 | 1.32E-01 | 0.53 | (0.48 - | 0.57) |  |
|  | *16* | 1.10 | (0.94 - | 1.28) | 1.37 | 2.41E-01 | 0.51 | (0.47 - | 0.56) |  |
|  | *32* | 1.06 | (0.91 - | 1.23) | 0.49 | 4.85E-01 | 0.51 | (0.46 - | 0.55) |  |
|  | *64* | 1.03 | (0.89 - | 1.20) | 0.19 | 6.65E-01 | 0.51 | (0.46 - | 0.55) |  |
| *Complexity* | *1* | 0.72 | (0.62 - | 0.85) | 16.67 | 4.44E-05 | 0.41 | (0.37 - | 0.46) |  |
|  | *2* | 0.78 | (0.67 - | 0.91) | 11.08 | 8.73E-04 | 0.42 | (0.38 - | 0.47) |  |
|  | *4* | 0.87 | (0.75 - | 1.00) | 4.06 | 4.39E-02 | 0.45 | (0.40 - | 0.49) |  |
|  | *6* | 0.90 | (0.79 - | 1.04) | 1.98 | 1.60E-01 | 0.45 | (0.41 - | 0.50) |  |
|  | *8* | 0.92 | (0.80 - | 1.06) | 1.41 | 2.35E-01 | 0.45 | (0.41 - | 0.50) |  |
|  | *16* | 0.98 | (0.85 - | 1.13) | 0.07 | 7.89E-01 | 0.48 | (0.43 - | 0.52) |  |
|  | *32* | 1.06 | (0.92 - | 1.23) | 0.65 | 4.19E-01 | 0.51 | (0.46 - | 0.55) |  |
|  | *64* | 1.06 | (0.90 - | 1.25) | 0.53 | 4.68E-01 | 0.51 | (0.46 - | 0.55) |  |
| *Contrast* | *1* | 0.76 | (0.65 - | 0.90) | 10.84 | 9.93E-04 | 0.42 | (0.37 - | 0.47) |  |
|  | *2* | 0.84 | (0.73 - | 0.98) | 5.14 | 2.33E-02 | 0.43 | (0.39 - | 0.48) |  |
|  | *4* | 0.92 | (0.80 - | 1.06) | 1.28 | 2.57E-01 | 0.45 | (0.40 - | 0.49) |  |
|  | *6* | 0.94 | (0.82 - | 1.09) | 0.64 | 4.23E-01 | 0.46 | (0.42 - | 0.51) |  |
|  | *8* | 0.96 | (0.83 - | 1.11) | 0.32 | 5.73E-01 | 0.46 | (0.42 - | 0.51) |  |
|  | *16* | 1.00 | (0.87 - | 1.16) | 0.00 | 9.50E-01 | 0.48 | (0.44 - | 0.53) |  |
|  | *32* | 1.07 | (0.92 - | 1.25) | 0.80 | 3.70E-01 | 0.51 | (0.46 - | 0.55) |  |
|  | *64* | 1.11 | (0.94 - | 1.31) | 1.61 | 2.04E-01 | 0.52 | (0.48 - | 0.56) |  |
| *Correlation* | *1* | 1.31 | (1.11 - | 1.55) | 10.90 | 9.59E-04 | 0.58 | (0.53 - | 0.62) |  |
|  | *2* | 1.19 | (1.02 - | 1.38) | 5.21 | 2.24E-02 | 0.57 | (0.52 - | 0.62) |  |
|  | *4* | 1.09 | (0.94 - | 1.25) | 1.32 | 2.51E-01 | 0.55 | (0.51 - | 0.60) |  |
|  | *6* | 1.06 | (0.92 - | 1.22) | 0.68 | 4.11E-01 | 0.54 | (0.49 - | 0.58) |  |
|  | *8* | 1.04 | (0.91 - | 1.20) | 0.36 | 5.49E-01 | 0.54 | (0.49 - | 0.58) |  |
|  | *16* | 1.00 | (0.87 - | 1.15) | 0.00 | 9.88E-01 | 0.52 | (0.48 - | 0.56) |  |
|  | *32* | 0.94 | (0.81 - | 1.09) | 0.62 | 4.31E-01 | 0.49 | (0.45 - | 0.54) |  |
|  | *64* | 0.92 | (0.78 - | 1.08) | 1.04 | 3.07E-01 | 0.47 | (0.43 - | 0.52) |  |
| *Dissimilarity* | *1* | 0.74 | (0.63 - | 0.86) | 15.02 | 1.06E-04 | 0.41 | (0.37 - | 0.46) |  |
|  | *2* | 0.82 | (0.71 - | 0.95) | 7.45 | 6.34E-03 | 0.43 | (0.38 - | 0.48) |  |
|  | *4* | 0.89 | (0.78 - | 1.03) | 2.44 | 1.18E-01 | 0.45 | (0.40 - | 0.50) |  |
|  | *6* | 0.92 | (0.80 - | 1.06) | 1.25 | 2.63E-01 | 0.47 | (0.42 - | 0.51) |  |
|  | *8* | 0.94 | (0.82 - | 1.09) | 0.66 | 4.18E-01 | 0.46 | (0.42 - | 0.51) |  |
|  | *16* | 1.01 | (0.88 - | 1.17) | 0.03 | 8.62E-01 | 0.48 | (0.44 - | 0.53) |  |
|  | *32* | 1.11 | (0.95 - | 1.30) | 1.70 | 1.92E-01 | 0.51 | (0.46 - | 0.55) |  |
|  | *64* | 1.16 | (0.98 - | 1.37) | 3.04 | 8.10E-02 | 0.53 | (0.49 - | 0.58) |  |
| *Energy* | *1* | 1.39 | (1.20 - | 1.61) | 19.33 | 1.10E-05 | 0.60 | (0.55 - | 0.64) |  |
|  | *2* | 1.27 | (1.10 - | 1.47) | 11.08 | 8.73E-04 | 0.58 | (0.53 - | 0.62) |  |
|  | *4* | 1.17 | (1.02 - | 1.35) | 4.84 | 2.78E-02 | 0.56 | (0.51 - | 0.61) |  |
|  | *6* | 1.13 | (0.98 - | 1.30) | 2.96 | 8.54E-02 | 0.55 | (0.50 - | 0.60) |  |
|  | *8* | 1.10 | (0.96 - | 1.27) | 1.88 | 1.70E-01 | 0.55 | (0.51 - | 0.60) |  |
|  | *16* | 1.01 | (0.87 - | 1.17) | 0.01 | 9.13E-01 | 0.53 | (0.49 - | 0.58) |  |
|  | *32* | 0.90 | (0.77 - | 1.06) | 1.62 | 2.03E-01 | 0.50 | (0.45 - | 0.54) |  |
|  | *64* | 0.87 | (0.74 - | 1.03) | 2.50 | 1.14E-01 | 0.48 | (0.43 - | 0.52) |  |
| *Entropy* | *1* | 0.73 | (0.63 - | 0.85) | 17.20 | 3.36E-05 | 0.41 | (0.36 - | 0.45) |  |
|  | *2* | 0.80 | (0.69 - | 0.93) | 9.33 | 2.26E-03 | 0.42 | (0.38 - | 0.47) |  |
|  | *4* | 0.87 | (0.76 - | 1.00) | 3.74 | 5.30E-02 | 0.44 | (0.40 - | 0.49) |  |
|  | *6* | 0.90 | (0.78 - | 1.03) | 2.29 | 1.30E-01 | 0.46 | (0.42 - | 0.51) |  |
|  | *8* | 0.92 | (0.79 - | 1.06) | 1.44 | 2.30E-01 | 0.46 | (0.42 - | 0.51) |  |
|  | *16* | 0.99 | (0.85 - | 1.14) | 0.03 | 8.64E-01 | 0.48 | (0.44 - | 0.53) |  |
|  | *32* | 1.09 | (0.93 - | 1.28) | 1.24 | 2.66E-01 | 0.50 | (0.46 - | 0.54) |  |
|  | *64* | 1.15 | (0.97 - | 1.36) | 2.80 | 9.45E-02 | 0.53 | (0.48 - | 0.57) |  |
| *GLN* | *16* | 1.09 | (0.93 - | 1.28) | 1.24 | 2.66E-01 | 0.51 | (0.47 - | 0.56) |  |
|  | *32* | 1.07 | (0.91 - | 1.26) | 0.67 | 4.14E-01 | 0.49 | (0.44 - | 0.54) |  |
|  | *64* | 1.04 | (0.88 - | 1.24) | 0.24 | 6.24E-01 | 0.50 | (0.45 - | 0.54) |  |
| *GLV* | *1* | 1.00 | (0.87 - | 1.16) | 0.00 | 9.76E-01 | 0.52 | (0.47 - | 0.56) |  |
|  | *2* | 1.00 | (0.87 - | 1.15) | 0.00 | 9.73E-01 | 0.50 | (0.46 - | 0.54) |  |
|  | *4* | 1.07 | (0.93 - | 1.23) | 0.80 | 3.72E-01 | 0.51 | (0.47 - | 0.56) |  |
|  | *6* | 1.10 | (0.95 - | 1.27) | 1.62 | 2.03E-01 | 0.53 | (0.48 - | 0.58) |  |
|  | *8* | 1.14 | (0.98 - | 1.32) | 3.11 | 7.80E-02 | 0.53 | (0.48 - | 0.57) |  |
|  | *16* | 1.23 | (1.06 - | 1.44) | 7.19 | 7.32E-03 | 0.54 | (0.49 - | 0.58) |  |
|  | *32* | 1.31 | (1.11 - | 1.55) | 10.89 | 9.65E-04 | 0.55 | (0.51 - | 0.59) |  |
|  | *64* | 1.41 | (1.19 - | 1.68) | 15.84 | 6.89E-05 | 0.58 | (0.53 - | 0.62) |  |
| *HGRE* | *1* | 1.21 | (1.05 - | 1.40) | 6.86 | 8.80E-03 | 0.57 | (0.52 - | 0.61) |  |
|  | *2* | 1.18 | (1.02 - | 1.36) | 4.85 | 2.77E-02 | 0.55 | (0.51 - | 0.60) |  |
|  | *4* | 1.13 | (0.98 - | 1.31) | 2.75 | 9.70E-02 | 0.54 | (0.50 - | 0.59) |  |
|  | *6* | 1.12 | (0.97 - | 1.29) | 2.18 | 1.40E-01 | 0.54 | (0.49 - | 0.58) |  |
|  | *8* | 1.10 | (0.94 - | 1.27) | 1.43 | 2.32E-01 | 0.53 | (0.48 - | 0.57) |  |
|  | *16* | 1.07 | (0.92 - | 1.25) | 0.76 | 3.84E-01 | 0.52 | (0.48 - | 0.56) |  |
|  | *32* | 1.06 | (0.90 - | 1.25) | 0.51 | 4.74E-01 | 0.51 | (0.46 - | 0.55) |  |
|  | *64* | 1.03 | (0.87 - | 1.22) | 0.10 | 7.47E-01 | 0.50 | (0.45 - | 0.54) |  |
| *HGZE* | *1* | 1.22 | (1.06 - | 1.39) | 7.88 | 5.00E-03 | 0.58 | (0.54 - | 0.63) |  |
|  | *2* | 1.19 | (1.04 - | 1.36) | 6.63 | 1.00E-02 | 0.57 | (0.53 - | 0.62) |  |
|  | *4* | 1.13 | (0.99 - | 1.29) | 3.47 | 6.25E-02 | 0.54 | (0.50 - | 0.59) |  |
|  | *6* | 1.09 | (0.96 - | 1.24) | 1.88 | 1.71E-01 | 0.54 | (0.49 - | 0.58) |  |
|  | *8* | 1.06 | (0.94 - | 1.21) | 0.87 | 3.51E-01 | 0.53 | (0.49 - | 0.58) |  |
|  | *16* | 1.02 | (0.88 - | 1.17) | 0.06 | 8.10E-01 | 0.50 | (0.46 - | 0.54) |  |
|  | *32* | 0.95 | (0.82 - | 1.10) | 0.49 | 4.82E-01 | 0.49 | (0.45 - | 0.54) |  |
|  | *64* | 0.90 | (0.78 - | 1.05) | 1.86 | 1.72E-01 | 0.47 | (0.43 - | 0.52) |  |
| *Homogeneity* | *1* | 1.37 | (1.18 - | 1.60) | 17.19 | 3.38E-05 | 0.59 | (0.54 - | 0.63) |  |
|  | *2* | 1.24 | (1.07 - | 1.44) | 8.58 | 3.40E-03 | 0.57 | (0.53 - | 0.62) |  |
|  | *4* | 1.14 | (0.99 - | 1.31) | 3.19 | 7.39E-02 | 0.55 | (0.50 - | 0.60) |  |
|  | *6* | 1.10 | (0.95 - | 1.26) | 1.73 | 1.89E-01 | 0.54 | (0.49 - | 0.58) |  |
|  | *8* | 1.07 | (0.93 - | 1.24) | 0.98 | 3.22E-01 | 0.54 | (0.49 - | 0.58) |  |
|  | *16* | 0.99 | (0.85 - | 1.14) | 0.04 | 8.48E-01 | 0.52 | (0.47 - | 0.56) |  |
|  | *32* | 0.89 | (0.75 - | 1.04) | 2.25 | 1.34E-01 | 0.49 | (0.45 - | 0.54) |  |
|  | *64* | 0.85 | (0.71 - | 1.00) | 3.84 | 5.01E-02 | 0.46 | (0.42 - | 0.50) |  |
| *LGRE* | *8* | 1.13 | (0.98 - | 1.30) | 2.68 | 1.02E-01 | 0.53 | (0.49 - | 0.58) |  |
|  | *16* | 1.08 | (0.93 - | 1.26) | 1.05 | 3.06E-01 | 0.52 | (0.47 - | 0.56) |  |
|  | *32* | 1.04 | (0.89 - | 1.22) | 0.28 | 6.00E-01 | 0.50 | (0.46 - | 0.55) |  |
|  | *64* | 1.03 | (0.87 - | 1.21) | 0.10 | 7.48E-01 | 0.49 | (0.45 - | 0.54) |  |
| *LGZE* | *2* | 1.23 | (1.08 - | 1.40) | 9.77 | 1.77E-03 | 0.59 | (0.55 - | 0.63) |  |
|  | *4* | 1.21 | (1.08 - | 1.35) | 10.96 | 9.32E-04 | 0.55 | (0.51 - | 0.60) |  |
|  | *6* | 1.17 | (1.05 - | 1.31) | 9.02 | 2.67E-03 | 0.54 | (0.50 - | 0.59) |  |
|  | *8* | 1.15 | (1.04 - | 1.28) | 6.96 | 8.34E-03 | 0.53 | (0.49 - | 0.58) |  |
|  | *16* | 1.11 | (1.00 - | 1.24) | 3.87 | 4.90E-02 | 0.50 | (0.46 - | 0.54) |  |
|  | *32* | 1.02 | (0.88 - | 1.17) | 0.05 | 8.17E-01 | 0.50 | (0.45 - | 0.54) |  |
|  | *64* | 0.90 | (0.77 - | 1.05) | 1.82 | 1.77E-01 | 0.47 | (0.43 - | 0.52) |  |
| *LRE* | *1* | 1.17 | (1.04 - | 1.32) | 6.28 | 1.22E-02 | 0.58 | (0.54 - | 0.63) |  |
|  | *2* | 1.16 | (1.02 - | 1.31) | 5.35 | 2.07E-02 | 0.58 | (0.54 - | 0.63) |  |
|  | *4* | 1.13 | (0.99 - | 1.28) | 3.29 | 6.97E-02 | 0.55 | (0.50 - | 0.59) |  |
|  | *6* | 1.11 | (0.97 - | 1.26) | 2.18 | 1.40E-01 | 0.55 | (0.50 - | 0.59) |  |
|  | *8* | 1.08 | (0.95 - | 1.24) | 1.27 | 2.59E-01 | 0.54 | (0.49 - | 0.58) |  |
|  | *16* | 1.00 | (0.86 - | 1.15) | 0.00 | 9.86E-01 | 0.51 | (0.47 - | 0.56) |  |
|  | *32* | 0.91 | (0.78 - | 1.06) | 1.46 | 2.27E-01 | 0.50 | (0.46 - | 0.54) |  |
|  | *64* | 0.85 | (0.73 - | 1.00) | 3.96 | 4.65E-02 | 0.46 | (0.42 - | 0.50) |  |
| *LRHGE* | *1* | 1.16 | (1.03 - | 1.31) | 5.90 | 1.51E-02 | 0.57 | (0.52 - | 0.62) |  |
|  | *2* | 1.15 | (1.02 - | 1.30) | 4.85 | 2.77E-02 | 0.56 | (0.51 - | 0.60) |  |
|  | *4* | 1.12 | (0.98 - | 1.27) | 2.82 | 9.30E-02 | 0.55 | (0.51 - | 0.60) |  |
|  | *6* | 1.09 | (0.96 - | 1.25) | 1.66 | 1.97E-01 | 0.54 | (0.50 - | 0.59) |  |
|  | *8* | 1.07 | (0.93 - | 1.22) | 0.82 | 3.66E-01 | 0.54 | (0.49 - | 0.58) |  |
|  | *16* | 0.98 | (0.84 - | 1.13) | 0.11 | 7.41E-01 | 0.52 | (0.47 - | 0.56) |  |
|  | *32* | 0.89 | (0.76 - | 1.04) | 2.32 | 1.28E-01 | 0.49 | (0.45 - | 0.54) |  |
|  | *64* | 0.83 | (0.70 - | 0.97) | 5.70 | 1.70E-02 | 0.45 | (0.40 - | 0.49) |  |
| *LRLGE* | *1* | 1.18 | (1.04 - | 1.34) | 6.18 | 1.29E-02 | 0.56 | (0.51 - | 0.61) |  |
|  | *2* | 1.17 | (1.03 - | 1.33) | 5.50 | 1.90E-02 | 0.56 | (0.51 - | 0.60) |  |
|  | *4* | 1.13 | (0.99 - | 1.29) | 3.33 | 6.79E-02 | 0.55 | (0.50 - | 0.59) |  |
|  | *6* | 1.11 | (0.97 - | 1.27) | 2.34 | 1.26E-01 | 0.54 | (0.49 - | 0.58) |  |
|  | *8* | 1.09 | (0.95 - | 1.25) | 1.48 | 2.24E-01 | 0.52 | (0.48 - | 0.57) |  |
|  | *16* | 1.01 | (0.88 - | 1.17) | 0.03 | 8.56E-01 | 0.49 | (0.45 - | 0.54) |  |
|  | *32* | 0.94 | (0.81 - | 1.09) | 0.75 | 3.87E-01 | 0.48 | (0.44 - | 0.52) |  |
|  | *64* | 0.91 | (0.77 - | 1.06) | 1.45 | 2.29E-01 | 0.45 | (0.41 - | 0.50) |  |
| *LZE* | *1* | 1.21 | (1.07 - | 1.37) | 9.08 | 2.59E-03 | 0.59 | (0.54 - | 0.63) |  |
|  | *2* | 1.18 | (1.04 - | 1.34) | 6.95 | 8.38E-03 | 0.57 | (0.53 - | 0.62) |  |
|  | *4* | 1.13 | (1.02 - | 1.27) | 5.01 | 2.52E-02 | 0.55 | (0.50 - | 0.59) |  |
|  | *6* | 1.11 | (1.00 - | 1.23) | 3.58 | 5.86E-02 | 0.54 | (0.49 - | 0.58) |  |
|  | *8* | 1.08 | (0.97 - | 1.21) | 1.98 | 1.59E-01 | 0.53 | (0.48 - | 0.58) |  |
|  | *16* | 0.97 | (0.83 - | 1.13) | 0.21 | 6.51E-01 | 0.51 | (0.46 - | 0.55) |  |
|  | *32* | 0.92 | (0.79 - | 1.09) | 0.95 | 3.29E-01 | 0.48 | (0.44 - | 0.52) |  |
|  | *64* | 0.94 | (0.80 - | 1.10) | 0.57 | 4.52E-01 | 0.43 | (0.39 - | 0.48) |  |
| *LZHGE* | *1* | 1.20 | (1.06 - | 1.35) | 8.15 | 4.30E-03 | 0.58 | (0.54 - | 0.63) |  |
|  | *2* | 1.17 | (1.03 - | 1.33) | 6.49 | 1.09E-02 | 0.57 | (0.52 - | 0.61) |  |
|  | *4* | 1.13 | (1.02 - | 1.27) | 5.13 | 2.36E-02 | 0.53 | (0.49 - | 0.58) |  |
|  | *6* | 1.11 | (1.00 - | 1.23) | 3.72 | 5.38E-02 | 0.53 | (0.49 - | 0.58) |  |
|  | *8* | 1.09 | (0.97 - | 1.21) | 2.09 | 1.48E-01 | 0.53 | (0.48 - | 0.58) |  |
|  | *16* | 0.97 | (0.84 - | 1.13) | 0.14 | 7.09E-01 | 0.52 | (0.47 - | 0.56) |  |
|  | *32* | 0.92 | (0.78 - | 1.08) | 1.14 | 2.86E-01 | 0.49 | (0.44 - | 0.53) |  |
|  | *64* | 0.93 | (0.79 - | 1.09) | 0.80 | 3.71E-01 | 0.46 | (0.41 - | 0.50) |  |
| *LZLGE* | *1* | 1.24 | (1.09 - | 1.41) | 10.70 | 1.07E-03 | 0.59 | (0.54 - | 0.64) |  |
|  | *2* | 1.20 | (1.06 - | 1.37) | 8.11 | 4.40E-03 | 0.58 | (0.53 - | 0.62) |  |
|  | *4* | 1.15 | (1.02 - | 1.29) | 5.38 | 2.04E-02 | 0.54 | (0.50 - | 0.59) |  |
|  | *6* | 1.11 | (0.99 - | 1.25) | 3.32 | 6.86E-02 | 0.54 | (0.49 - | 0.58) |  |
|  | *8* | 1.09 | (0.97 - | 1.23) | 1.96 | 1.61E-01 | 0.53 | (0.48 - | 0.58) |  |
|  | *16* | 0.94 | (0.80 - | 1.11) | 0.58 | 4.46E-01 | 0.51 | (0.47 - | 0.56) |  |
|  | *32* | 0.93 | (0.79 - | 1.09) | 0.92 | 3.37E-01 | 0.48 | (0.44 - | 0.52) |  |
|  | *64* | 0.97 | (0.83 - | 1.13) | 0.16 | 6.90E-01 | 0.43 | (0.39 - | 0.48) |  |
| *RLN* | *8* | 1.08 | (0.91 - | 1.28) | 0.72 | 3.96E-01 | 0.52 | (0.47 - | 0.56) |  |
|  | *16* | 1.09 | (0.92 - | 1.30) | 1.09 | 2.97E-01 | 0.52 | (0.48 - | 0.57) |  |
|  | *32* | 1.10 | (0.93 - | 1.30) | 1.16 | 2.80E-01 | 0.53 | (0.48 - | 0.57) |  |
|  | *64* | 1.06 | (0.90 - | 1.25) | 0.46 | 4.99E-01 | 0.51 | (0.47 - | 0.55) |  |
| *RLV* | *1* | 1.25 | (1.07 - | 1.46) | 7.86 | 5.05E-03 | 0.57 | (0.53 - | 0.61) |  |
|  | *2* | 1.33 | (1.14 - | 1.56) | 12.83 | 3.42E-04 | 0.59 | (0.54 - | 0.63) |  |
|  | *4* | 1.34 | (1.14 - | 1.58) | 13.29 | 2.67E-04 | 0.58 | (0.53 - | 0.62) |  |
|  | *6* | 1.35 | (1.14 - | 1.59) | 12.89 | 3.30E-04 | 0.59 | (0.54 - | 0.63) |  |
|  | *8* | 1.39 | (1.18 - | 1.65) | 15.46 | 8.42E-05 | 0.58 | (0.54 - | 0.62) |  |
|  | *16* | 1.38 | (1.17 - | 1.64) | 14.71 | 1.25E-04 | 0.57 | (0.52 - | 0.61) |  |
|  | *32* | 1.37 | (1.15 - | 1.62) | 13.67 | 2.18E-04 | 0.57 | (0.53 - | 0.62) |  |
|  | *64* | 1.44 | (1.21 - | 1.72) | 18.03 | 2.17E-05 | 0.59 | (0.55 - | 0.63) |  |
| *RP* | *1* | 0.72 | (0.60 - | 0.86) | 13.69 | 2.16E-04 | 0.42 | (0.37 - | 0.46) |  |
|  | *2* | 0.78 | (0.65 - | 0.93) | 8.50 | 3.55E-03 | 0.44 | (0.40 - | 0.48) |  |
|  | *4* | 0.83 | (0.71 - | 0.98) | 5.00 | 2.54E-02 | 0.45 | (0.41 - | 0.50) |  |
|  | *6* | 0.85 | (0.72 - | 1.00) | 4.15 | 4.16E-02 | 0.46 | (0.42 - | 0.51) |  |
|  | *8* | 0.86 | (0.73 - | 1.02) | 3.27 | 7.04E-02 | 0.47 | (0.42 - | 0.51) |  |
|  | *16* | 0.90 | (0.77 - | 1.06) | 1.51 | 2.19E-01 | 0.50 | (0.45 - | 0.54) |  |
|  | *32* | 0.95 | (0.80 - | 1.12) | 0.43 | 5.10E-01 | 0.52 | (0.47 - | 0.56) |  |
|  | *64* | 1.01 | (0.85 - | 1.20) | 0.01 | 9.35E-01 | 0.51 | (0.47 - | 0.56) |  |
| *SRE* | *6* | 1.11 | (0.94 - | 1.31) | 1.60 | 2.05E-01 | 0.52 | (0.47 - | 0.56) |  |
|  | *8* | 1.09 | (0.92 - | 1.29) | 1.07 | 3.00E-01 | 0.52 | (0.48 - | 0.57) |  |
|  | *16* | 1.09 | (0.93 - | 1.29) | 1.09 | 2.96E-01 | 0.52 | (0.48 - | 0.57) |  |
|  | *32* | 1.09 | (0.92 - | 1.29) | 1.00 | 3.18E-01 | 0.52 | (0.48 - | 0.56) |  |
|  | *64* | 1.05 | (0.89 - | 1.25) | 0.35 | 5.52E-01 | 0.51 | (0.46 - | 0.55) |  |
| *SRHGE* | *1* | 1.18 | (1.03 - | 1.36) | 5.23 | 2.23E-02 | 0.56 | (0.51 - | 0.60) |  |
|  | *2* | 1.12 | (0.97 - | 1.30) | 2.29 | 1.31E-01 | 0.53 | (0.48 - | 0.57) |  |
|  | *4* | 1.08 | (0.93 - | 1.26) | 0.96 | 3.27E-01 | 0.52 | (0.47 - | 0.56) |  |
|  | *6* | 1.08 | (0.92 - | 1.25) | 0.86 | 3.53E-01 | 0.51 | (0.47 - | 0.56) |  |
|  | *8* | 1.06 | (0.90 - | 1.23) | 0.45 | 5.00E-01 | 0.51 | (0.46 - | 0.55) |  |
|  | *16* | 1.07 | (0.91 - | 1.26) | 0.66 | 4.15E-01 | 0.51 | (0.47 - | 0.56) |  |
|  | *32* | 1.09 | (0.92 - | 1.28) | 0.95 | 3.30E-01 | 0.52 | (0.48 - | 0.57) |  |
|  | *64* | 1.03 | (0.87 - | 1.22) | 0.12 | 7.29E-01 | 0.51 | (0.47 - | 0.56) |  |
| *SRLGE* | *16* | 1.10 | (0.95 - | 1.28) | 1.57 | 2.10E-01 | 0.53 | (0.48 - | 0.57) |  |
|  | *32* | 1.05 | (0.90 - | 1.24) | 0.40 | 5.28E-01 | 0.50 | (0.46 - | 0.55) |  |
|  | *64* | 1.04 | (0.88 - | 1.22) | 0.20 | 6.58E-01 | 0.49 | (0.45 - | 0.54) |  |
| *SZE* | *1* | 1.23 | (1.07 - | 1.41) | 8.47 | 3.61E-03 | 0.58 | (0.53 - | 0.62) |  |
|  | *2* | 1.18 | (1.03 - | 1.35) | 5.86 | 1.55E-02 | 0.56 | (0.52 - | 0.61) |  |
|  | *4* | 1.13 | (0.99 - | 1.28) | 3.13 | 7.69E-02 | 0.53 | (0.49 - | 0.58) |  |
|  | *6* | 1.11 | (0.98 - | 1.25) | 2.57 | 1.09E-01 | 0.53 | (0.49 - | 0.58) |  |
|  | *8* | 1.08 | (0.96 - | 1.23) | 1.51 | 2.20E-01 | 0.51 | (0.47 - | 0.56) |  |
|  | *16* | 1.00 | (0.87 - | 1.15) | 0.00 | 9.93E-01 | 0.51 | (0.46 - | 0.55) |  |
|  | *32* | 0.91 | (0.78 - | 1.06) | 1.49 | 2.23E-01 | 0.46 | (0.42 - | 0.51) |  |
|  | *64* | 0.88 | (0.75 - | 1.03) | 2.44 | 1.18E-01 | 0.45 | (0.41 - | 0.50) |  |
| *SZHGE* | *1* | 1.21 | (1.06 - | 1.39) | 7.81 | 5.21E-03 | 0.58 | (0.53 - | 0.62) |  |
|  | *2* | 1.17 | (1.03 - | 1.33) | 5.56 | 1.84E-02 | 0.55 | (0.51 - | 0.60) |  |
|  | *4* | 1.11 | (0.98 - | 1.26) | 2.63 | 1.05E-01 | 0.53 | (0.48 - | 0.57) |  |
|  | *6* | 1.09 | (0.96 - | 1.23) | 1.73 | 1.89E-01 | 0.53 | (0.49 - | 0.58) |  |
|  | *8* | 1.04 | (0.92 - | 1.19) | 0.43 | 5.14E-01 | 0.51 | (0.46 - | 0.55) |  |
|  | *16* | 0.96 | (0.82 - | 1.12) | 0.33 | 5.63E-01 | 0.50 | (0.45 - | 0.54) |  |
|  | *32* | 0.91 | (0.78 - | 1.06) | 1.57 | 2.10E-01 | 0.48 | (0.44 - | 0.53) |  |
|  | *64* | 0.95 | (0.82 - | 1.10) | 0.46 | 4.98E-01 | 0.48 | (0.44 - | 0.53) |  |
| *SZLGE* | *2* | 1.21 | (1.06 - | 1.39) | 7.98 | 4.74E-03 | 0.58 | (0.53 - | 0.62) |  |
|  | *4* | 1.20 | (1.06 - | 1.34) | 9.48 | 2.07E-03 | 0.55 | (0.50 - | 0.59) |  |
|  | *6* | 1.18 | (1.06 - | 1.31) | 9.46 | 2.10E-03 | 0.54 | (0.49 - | 0.58) |  |
|  | *8* | 1.17 | (1.06 - | 1.30) | 9.27 | 2.33E-03 | 0.53 | (0.48 - | 0.57) |  |
|  | *16* | 1.10 | (1.00 - | 1.22) | 4.06 | 4.38E-02 | 0.50 | (0.46 - | 0.55) |  |
|  | *32* | 1.01 | (0.88 - | 1.16) | 0.03 | 8.66E-01 | 0.50 | (0.46 - | 0.54) |  |
|  | *64* | 0.96 | (0.83 - | 1.11) | 0.33 | 5.64E-01 | 0.49 | (0.44 - | 0.53) |  |
| *Strength* | *1* | 1.22 | (1.06 - | 1.41) | 7.29 | 6.94E-03 | 0.58 | (0.53 - | 0.62) |  |
|  | *2* | 1.19 | (1.03 - | 1.38) | 5.52 | 1.88E-02 | 0.56 | (0.51 - | 0.60) |  |
|  | *4* | 1.15 | (0.99 - | 1.34) | 3.48 | 6.22E-02 | 0.54 | (0.49 - | 0.58) |  |
|  | *6* | 1.14 | (0.98 - | 1.33) | 2.88 | 8.99E-02 | 0.53 | (0.49 - | 0.58) |  |
|  | *8* | 1.13 | (0.97 - | 1.31) | 2.27 | 1.32E-01 | 0.53 | (0.48 - | 0.57) |  |
|  | *16* | 1.10 | (0.94 - | 1.28) | 1.38 | 2.40E-01 | 0.51 | (0.47 - | 0.56) |  |
|  | *32* | 1.06 | (0.91 - | 1.23) | 0.51 | 4.75E-01 | 0.51 | (0.46 - | 0.55) |  |
|  | *64* | 1.04 | (0.89 - | 1.20) | 0.20 | 6.53E-01 | 0.50 | (0.46 - | 0.55) |  |
| *SumAverage* | *1* | 1.06 | (0.93 - | 1.21) | 0.80 | 3.70E-01 | 0.50 | (0.45 - | 0.54) |  |
|  | *2* | 1.23 | (1.07 - | 1.41) | 9.11 | 2.55E-03 | 0.56 | (0.51 - | 0.60) |  |
|  | *4* | 1.24 | (1.08 - | 1.43) | 9.22 | 2.39E-03 | 0.58 | (0.53 - | 0.62) |  |
|  | *6* | 1.30 | (1.13 - | 1.49) | 14.24 | 1.61E-04 | 0.58 | (0.54 - | 0.62) |  |
|  | *8* | 1.35 | (1.17 - | 1.55) | 18.32 | 1.87E-05 | 0.57 | (0.52 - | 0.61) |  |
|  | *16* | 1.52 | (1.31 - | 1.77) | 31.26 | 2.25E-08 | 0.61 | (0.56 - | 0.65) |  |
|  | *32* | 1.52 | (1.31 - | 1.77) | 31.75 | 1.75E-08 | 0.61 | (0.56 - | 0.65) |  |
|  | *64* | 1.48 | (1.28 - | 1.71) | 29.07 | 6.98E-08 | 0.58 | (0.54 - | 0.63) |  |
| *Variance* | *1* | 1.07 | (0.93 - | 1.24) | 1.01 | 3.15E-01 | 0.52 | (0.48 - | 0.57) |  |
|  | *2* | 1.09 | (0.95 - | 1.25) | 1.43 | 2.32E-01 | 0.52 | (0.47 - | 0.56) |  |
|  | *4* | 1.05 | (0.92 - | 1.20) | 0.48 | 4.89E-01 | 0.52 | (0.47 - | 0.56) |  |
|  | *6* | 1.12 | (0.97 - | 1.29) | 2.29 | 1.30E-01 | 0.54 | (0.49 - | 0.58) |  |
|  | *8* | 1.17 | (1.01 - | 1.35) | 4.31 | 3.79E-02 | 0.53 | (0.49 - | 0.58) |  |
|  | *16* | 1.19 | (1.02 - | 1.38) | 5.30 | 2.14E-02 | 0.55 | (0.51 - | 0.59) |  |
|  | *32* | 1.34 | (1.15 - | 1.57) | 14.59 | 1.33E-04 | 0.58 | (0.53 - | 0.62) |  |
|  | *64* | 1.33 | (1.14 - | 1.54) | 14.37 | 1.51E-04 | 0.60 | (0.55 - | 0.64) |  |
| *ZP* | *1* | 0.75 | (0.61 - | 0.93) | 8.15 | 4.30E-03 | 0.42 | (0.37 - | 0.46) |  |
|  | *2* | 0.82 | (0.68 - | 1.01) | 4.37 | 3.65E-02 | 0.42 | (0.37 - | 0.47) |  |
|  | *4* | 0.93 | (0.79 - | 1.08) | 0.99 | 3.20E-01 | 0.45 | (0.40 - | 0.49) |  |
|  | *6* | 0.94 | (0.81 - | 1.09) | 0.67 | 4.12E-01 | 0.46 | (0.41 - | 0.51) |  |
|  | *8* | 0.95 | (0.82 - | 1.10) | 0.46 | 4.97E-01 | 0.48 | (0.44 - | 0.53) |  |
|  | *16* | 0.99 | (0.85 - | 1.14) | 0.04 | 8.48E-01 | 0.49 | (0.44 - | 0.53) |  |
|  | *32* | 1.04 | (0.90 - | 1.21) | 0.34 | 5.59E-01 | 0.53 | (0.49 - | 0.58) |  |
|  | *64* | 1.22 | (1.06 - | 1.41) | 7.67 | 5.62E-03 | 0.55 | (0.51 - | 0.59) |  |
| *ZSN* | *1* | 1.23 | (1.07 - | 1.41) | 8.44 | 3.68E-03 | 0.57 | (0.53 - | 0.62) |  |
|  | *2* | 1.16 | (1.02 - | 1.32) | 4.78 | 2.87E-02 | 0.55 | (0.50 - | 0.59) |  |
|  | *4* | 1.10 | (0.97 - | 1.25) | 2.27 | 1.32E-01 | 0.54 | (0.49 - | 0.58) |  |
|  | *6* | 1.09 | (0.97 - | 1.23) | 2.21 | 1.37E-01 | 0.52 | (0.48 - | 0.57) |  |
|  | *8* | 1.06 | (0.93 - | 1.20) | 0.77 | 3.79E-01 | 0.51 | (0.47 - | 0.56) |  |
|  | *16* | 0.95 | (0.82 - | 1.10) | 0.44 | 5.07E-01 | 0.50 | (0.45 - | 0.54) |  |
|  | *32* | 0.89 | (0.76 - | 1.04) | 2.38 | 1.23E-01 | 0.47 | (0.42 - | 0.51) |  |
|  | *64* | 0.93 | (0.80 - | 1.10) | 0.72 | 3.96E-01 | 0.47 | (0.42 - | 0.52) |  |
| *ZSV* | *1* | 1.26 | (1.10 - | 1.44) | 11.12 | 8.54E-04 | 0.58 | (0.54 - | 0.63) |  |
|  | *2* | 1.23 | (1.08 - | 1.40) | 9.04 | 2.64E-03 | 0.58 | (0.53 - | 0.62) |  |
|  | *4* | 1.17 | (1.03 - | 1.32) | 5.56 | 1.84E-02 | 0.55 | (0.50 - | 0.60) |  |
|  | *6* | 1.13 | (1.00 - | 1.28) | 3.79 | 5.16E-02 | 0.53 | (0.49 - | 0.58) |  |
|  | *8* | 1.11 | (0.98 - | 1.25) | 2.49 | 1.15E-01 | 0.53 | (0.48 - | 0.57) |  |
|  | *16* | 1.01 | (0.88 - | 1.17) | 0.03 | 8.66E-01 | 0.51 | (0.47 - | 0.55) |  |
|  | *32* | 0.89 | (0.76 - | 1.04) | 2.22 | 1.36E-01 | 0.47 | (0.42 - | 0.51) |  |
|  | *64* | 0.84 | (0.71 - | 0.99) | 4.69 | 3.03E-02 | 0.43 | (0.39 - | 0.47) |  |
| *Area* | *1* | 0.87 | (0.72 - | 1.04) | 2.42 | 1.20E-01 | 0.47 | (0.43 - | 0.52) |  |
| *ConvexArea* | *1* | 0.86 | (0.72 - | 1.03) | 2.59 | 1.08E-01 | 0.47 | (0.42 - | 0.51) |  |
| *Eccentricity* | *1* | 1.24 | (1.06 - | 1.46) | 7.11 | 7.67E-03 | 0.54 | (0.49 - | 0.59) |  |
| *Equivalent diameter* | *1* | 0.87 | (0.72 - | 1.04) | 2.44 | 1.19E-01 | 0.47 | (0.43 - | 0.52) |  |
| *Extent* | *1* | 0.96 | (0.83 - | 1.10) | 0.35 | 5.53E-01 | 0.48 | (0.43 - | 0.52) |  |
| *glszmGLN* | *2* | 1.20 | (1.05 - | 1.38) | 6.92 | 8.53E-03 | 0.58 | (0.53 - | 0.62) |  |
|  | *4* | 1.15 | (1.00 - | 1.32) | 4.09 | 4.32E-02 | 0.56 | (0.52 - | 0.61) |  |
|  | *6* | 1.12 | (0.98 - | 1.28) | 2.59 | 1.07E-01 | 0.56 | (0.52 - | 0.60) |  |
|  | *8* | 1.09 | (0.96 - | 1.24) | 1.65 | 1.99E-01 | 0.55 | (0.51 - | 0.60) |  |
|  | *16* | 1.06 | (0.93 - | 1.22) | 0.72 | 3.96E-01 | 0.52 | (0.48 - | 0.57) |  |
|  | *32* | 0.96 | (0.83 - | 1.12) | 0.27 | 6.04E-01 | 0.50 | (0.46 - | 0.54) |  |
|  | *64* | 0.89 | (0.76 - | 1.04) | 2.36 | 1.25E-01 | 0.45 | (0.41 - | 0.49) |  |
| *glszmGLV* | *1* | 1.13 | (0.96 - | 1.32) | 2.24 | 1.35E-01 | 0.53 | (0.48 - | 0.57) |  |
|  | *2* | 1.02 | (0.88 - | 1.18) | 0.04 | 8.42E-01 | 0.50 | (0.46 - | 0.54) |  |
|  | *4* | 0.98 | (0.84 - | 1.14) | 0.09 | 7.70E-01 | 0.49 | (0.45 - | 0.54) |  |
|  | *6* | 0.97 | (0.84 - | 1.13) | 0.14 | 7.05E-01 | 0.46 | (0.42 - | 0.50) |  |
|  | *8* | 0.93 | (0.80 - | 1.08) | 0.96 | 3.28E-01 | 0.45 | (0.41 - | 0.50) |  |
|  | *16* | 0.97 | (0.83 - | 1.13) | 0.17 | 6.79E-01 | 0.49 | (0.44 - | 0.53) |  |
|  | *32* | 1.06 | (0.91 - | 1.23) | 0.51 | 4.76E-01 | 0.52 | (0.48 - | 0.57) |  |
|  | *64* | 1.11 | (0.95 - | 1.30) | 1.61 | 2.04E-01 | 0.55 | (0.50 - | 0.59) |  |
| *Kurtosis* | *1* | 0.89 | (0.77 - | 1.03) | 2.62 | 1.06E-01 | 0.46 | (0.41 - | 0.50) |  |
| *Major axis length* | *1* | 0.99 | (0.82 - | 1.19) | 0.02 | 8.85E-01 | 0.50 | (0.45 - | 0.54) |  |
| *Mean* | *1* | 1.10 | (0.95 - | 1.26) | 1.62 | 2.03E-01 | 0.52 | (0.48 - | 0.56) |  |
| *Median* | *1* | 1.10 | (0.96 - | 1.27) | 1.91 | 1.68E-01 | 0.53 | (0.49 - | 0.57) |  |
| *Minor axis length* | *1* | 0.83 | (0.70 - | 0.99) | 4.61 | 3.17E-02 | 0.47 | (0.42 - | 0.51) |  |
| *Mode* | *1* | 1.07 | (0.93 - | 1.23) | 1.01 | 3.15E-01 | 0.52 | (0.47 - | 0.56) |  |
| *ngtdmContrast* | *1* | 0.73 | (0.62 - | 0.85) | 16.75 | 4.27E-05 | 0.40 | (0.36 - | 0.45) |  |
|  | *2* | 0.78 | (0.67 - | 0.91) | 11.17 | 8.33E-04 | 0.41 | (0.37 - | 0.46) |  |
|  | *4* | 0.86 | (0.75 - | 1.00) | 4.15 | 4.16E-02 | 0.44 | (0.40 - | 0.49) |  |
|  | *6* | 0.90 | (0.78 - | 1.04) | 2.03 | 1.54E-01 | 0.46 | (0.41 - | 0.50) |  |
|  | *8* | 0.92 | (0.80 - | 1.06) | 1.39 | 2.39E-01 | 0.46 | (0.42 - | 0.51) |  |
|  | *16* | 0.98 | (0.85 - | 1.13) | 0.09 | 7.61E-01 | 0.47 | (0.43 - | 0.52) |  |
|  | *32* | 1.06 | (0.92 - | 1.23) | 0.64 | 4.25E-01 | 0.50 | (0.45 - | 0.54) |  |
|  | *64* | 1.06 | (0.90 - | 1.25) | 0.55 | 4.56E-01 | 0.51 | (0.46 - | 0.55) |  |
| *Perimeter* | *1* | 0.91 | (0.77 - | 1.07) | 1.37 | 2.42E-01 | 0.46 | (0.41 - | 0.50) |  |
| *SD* | *1* | 1.32 | (1.13 - | 1.54) | 13.11 | 2.94E-04 | 0.57 | (0.52 - | 0.61) |  |
| *Skewness* | *1* | 1.01 | (0.88 - | 1.16) | 0.03 | 8.72E-01 | 0.51 | (0.47 - | 0.55) |  |
| *Solidity* | *1* | 1.03 | (0.87 - | 1.21) | 0.12 | 7.32E-01 | 0.53 | (0.48 - | 0.57) |  |

Standardized odds ratio: change in odds for a standard deviation (in controls) increase in predictors, adjusted for age and BMI; CI: confidence interval; mC: matched concordance index. Total number of observations (N) = 1051, including 264 cases and 787 controls.

Note: Some features at certain downsize factors are not presented because the conditional logistic regressions failed to complete in the statistical software used (SAS 9.4, “ERROR: All explanatory variables are dependent on the strata”).

The following features are based on NGTDM: busyness, coarseness, complexity, strength, ngtdmContrast.

The following features are based on GLCM: contrast, correlation, dissimilarity, energy, entropy, homogeneity, sum average, variance.

The following features are based on run-length matrix: GLN, GLV, HGRE, LGRE, LRE, LRHGE, LRLGE, RLN, RLV, RP, SRE, SRHGE, SRLGE.

The following features are based on GLSZM: HGZE, LGZE, LZE, LZHGE, LZLGE, SZE, SZHGE, SZLGE, ZP, ZSN, ZSV, glszmGLN, glszmGLV.

The following features are form based: area, convex area, eccentricity, equivalent diameter, extent, major axis length, minor axis length, perimeter, solidity.

The following features are moment based: kurtosis, mean, median, mode, SD, skewness.

Abbreviations:

- GLCM: gray-level co-occurrence matrix
- GLSZM: gray-level size zone matrix
- NGTDM: neighbourhood gray-tone difference matrix
- SD: standard deviation
- GLN: Gray-Level Nonuniformity
- GLV: Gray-Level Variance
- HGRE: High Gray-Level Run Emphasis
- HGZE: High Gray-Level Zone Emphasis
- LGRE: Low Gray-Level Run Emphasis
- LGZE: Low Gray-Level Zone Emphasis
- LRE: Long Run Emphasis
- LRHGE: Long Run High Gray-Level Emphasis
- LRLGE: Long Run Low Gray-Level Emphasis
- LZE: Large Zone Emphasis
- LZHGE: Large Zone High Gray-Level Emphasis
- LZLGE: Large Zone Low Gray-Level Emphasis
- RLN: Run-Length Nonuniformity
- RLV: Run-Length Variance
- RP: Run Percentage
- SRE: Short Run Emphasis
- SRHGE: Short Run High Gray-Level Emphasis
- SRLGE: Short Run Low Gray-Level Emphasis
- SZE: Small Zone Emphasis
- SZHGE: Small Zone High Gray-Level Emphasis
- SZLGE: Small Zone Low Gray-Level Emphasis
- ZP: Zone Percentage
- ZSN: Zone-Size Nonuniformity
- ZSV: Zone-Size Variance
